# Home Physiotherapy Using the Proprioceptive Neuromuscular Facilitation Concept in a Patient with Chronic Hemiplegia in a Rural Area: A Case Report

**DOI:** 10.3390/jcm14248913

**Published:** 2025-12-17

**Authors:** Tomasz Zwoliński, Kamila Gworys, Michał Licznerski, Katarzyna Zorena

**Affiliations:** 1 Faculty of Health, University WSB Merito Gdańsk, 80-266 Gdańsk, Poland; 2 Department of Physical Rehabilitation Medicine, Medical University of Łódź, 90-419 Łódź, Poland; kamila.gworys@umed.lodz.pl; 3 Faculty of Health Sciences, Medical University of Gdańsk, 80-210 Gdańsk, Poland; m-licznerski@gumed.edu.pl; 4 Department of Immunobiology and Environmental Microbiology, Medical University of Gdańsk, 80-210 Gdańsk, Poland; katarzyna.zorena@gumed.edu.pl

**Keywords:** proprioceptive neuromuscular facilitation (PNF), postural balance, gait, activities of daily living, quality of life, stroke rehabilitation, stroke, home-based rehabilitation

## Abstract

**Background/Objectives:** The growing population of stroke survivors living in rural communities, who require ongoing rehabilitation, highlights the need for effective, home-based rehabilitation methods. The aim of this study was to evaluate the potential benefits of Proprioceptive Neuromuscular Facilitation (PNF) on segmental and global motor function, gait performance, Activities of Daily Living (ADLs), and quality of life in a patient with chronic hemiplegia living at home. **Methods:** A 71-year-old woman, five years after an ischemic stroke of the left cerebral hemisphere, presented with severe right-sided hemiplegia and participated in a two-month home-based PNF rehabilitation programme consisting of 20 one-hour sessions delivered 2–3 times per week. To evaluate the effectiveness of the intervention, the following standardised outcome measures were used in given order: Fugl-Meyer Assessment of Lower Extremity (FMA-LE), Trunk Impairment Scale (TIS), Timed Up and Go Test (TUG), Rivermead Motor Assessment Scale (RMA), Barthel Scale (BS), and the Short Form Health Survey (SF-36). **Results:** Improvements were observed in lower limb motor function (FMA-LE), gait performance (TUG), and overall quality of life (SF-36). **Conclusions:** PNF-based physiotherapy delivered at home environment may provide meaningful benefits for patients living in rural areas, even years after stroke.

## 1. Introduction

Stroke remains the second leading cause of death and the third leading cause of combined death and disability [1]. Given increased life expectancy and the prevalence of unhealthy lifestyle factors, an increase in the incidence of stroke can be expected.

There is also a search for effective methods to improve the functional status of people with movement deficits following a stroke. One such method is Proprioceptive Neuromuscular Facilitation (PNF) [2,3]. The PNF approach distinguishes itself from other therapeutic approaches by using specific patterns and techniques that target the body’s proprioceptive system [4]. PNF is utilised in the rehabilitation of stroke patients because it enhances motor function by enhancing neuroplasticity and improving proprioceptive integration, resulting in improved movement control, muscle strength, and coordination [5]. It is a neuromuscular rehabilitation therapy that improves neuromuscular activation patterns, improving strength, postural control and movement coordination [6]. Moreover, specific movement patterns and proprioceptive stimulation applied within the PNF concept optimize sensory-motor processing the central nervous system, thereby promoting the reorganization of neural networks following brain damage [5]. PNF also uses functional, multiplanar movement patterns engaging synergist muscle groups, activating neuroplasticity mechanisms and sensory-motor integration, which is particularly important in post-stroke rehabilitation [6].

It is a widely used physical therapy treatment in various patient populations, including those with a wide range of neurological and musculoskeletal conditions [7,8].

PNF techniques are helpful in training activities of daily living, improving the patient’s ability to perform ADLs, and promoting independence in daily life. In addition, these exercises are unique because the patient must exert a significant amount of physical effort, yet they do not require any specialised medical equipment [9,10]. Moreover, specific movement patterns and proprioceptive stimulation applied within the PNF concept enhance the efficiency of stimulus transmission to the central nervous system, thereby promoting the reorganization of neural networks following brain damage. However, it is worth noting that preliminary research suggests that combining PNF with other modern technologies (e.g., robotic gait training, EMS, kinesiotaping) may produce better results than either intervention alone [11,12].

Many researchers emphasise the fact that balance disorders in people after stroke manifest themselves as asymmetry in shifting body weight towards the healthy side [13,14]. There are reports that PNF also positively affects stroke patients’ ability to maintain dynamic balance, walking efficiency, and reduces the fear of falling (FoF) [15,16].

Therefore, this study aimed to assess the impact of selected PNF techniques on a stroke patient during home physiotherapy in rural areas. As a result, the patient’s quality of life, functionality in everyday life, global and segmental motor performance, lower limb function, coordination, balance, somatosensory function, and gait function were evaluated.

## 2. Material and Methods

This case report presents a 71-year-old woman living in a rural area, five years after an ischemic stroke of the left cerebral hemisphere, presented with right-sided hemiplegia, VII cranial nerve palsy, motor aphasia, and dysarthria. The patient had previously worked as a school cleaner and is currently retired.

The TIDieR (Template for Intervention Description and Replication) [17] and CARE (Case Report) guidelines [18] were applied to enhance transparency and reporting rigor. Both checklists are provided in the Supplementary Materials Section [Table S1: TIDieR Checklist; Table S2: CARE Checklist].

The diagnosis was confirmed by computed tomography (CT) of the head, which revealed a hyperdense left middle cerebral artery, suggestive of acute arterial occlusion. A follow-up non-contrast CT demonstrated an established ischemic infarct within the left cerebral hemisphere, involving parts of the insula, lentiform nucleus, caudate nucleus, and the posterior limb of the internal capsule.

Patient’s comorbidities included hypertension, mild aortic regurgitation, paroxysmal atrial fibrillation, New York Heart Association (NYHA) Class II heart failure, varicose veins of the lower limbs, and obesity.

The rehabilitation program was delivered in the patient’s residence within a rural community in the Pomeranian Voivodeship, Poland, situated in the Vistula Żuławy region (population: 977).

**Figure 1:**
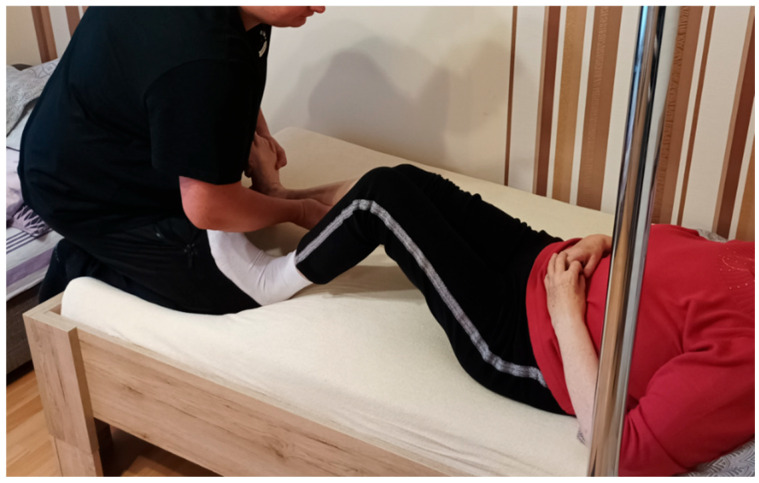
Resisted flexion of the non-affected lower limb to facilitate activation of extensor musculature in the hemiparetic lower limb using the Combination of Isotonics technique based on PNF principles.

**Figure 2:**
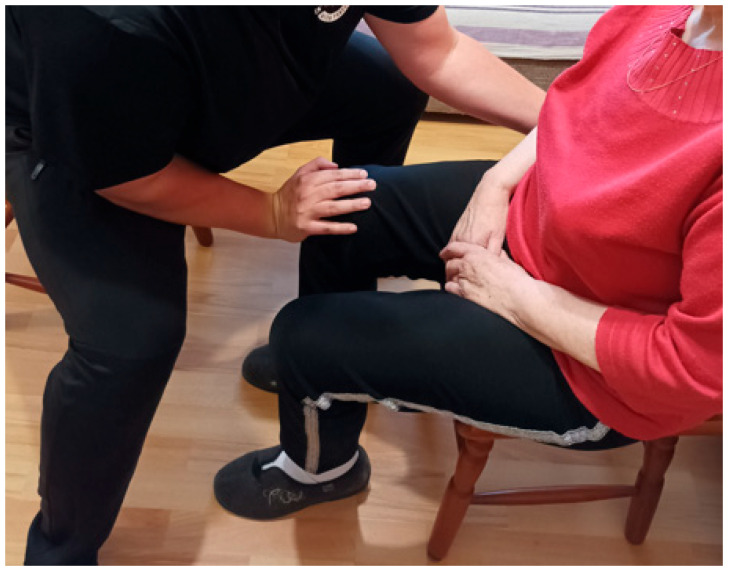
Guided forward and backward movement of the hemiparetic lower limb (knee) to teach correct initiation and limb use, using the Rhythmic Initiation technique from the PNF approach.

**Figure 3:**
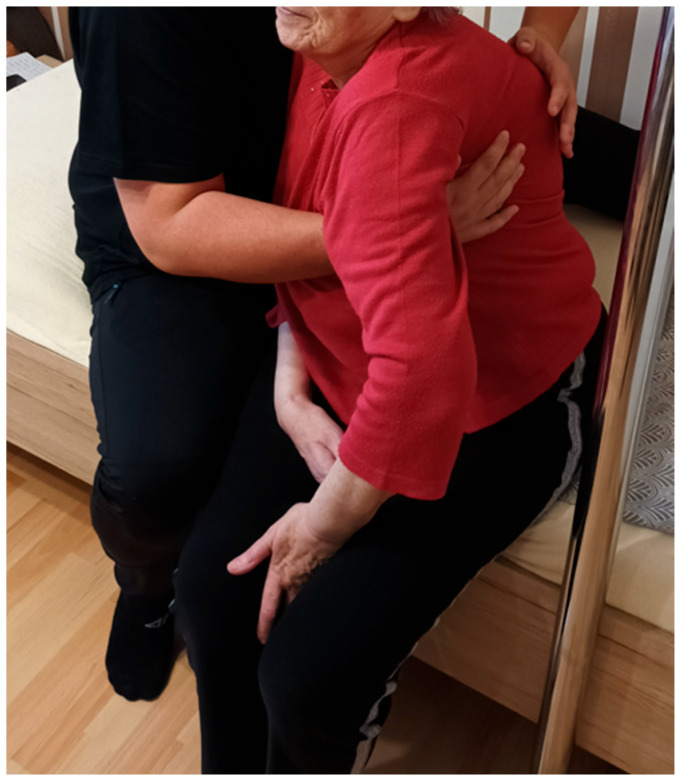
Resisted trunk rotation to both sides to strengthen the trunk and enhance rotational mobility using the Combination of Isotonics technique based on PNF principles.

**Figure 4:**
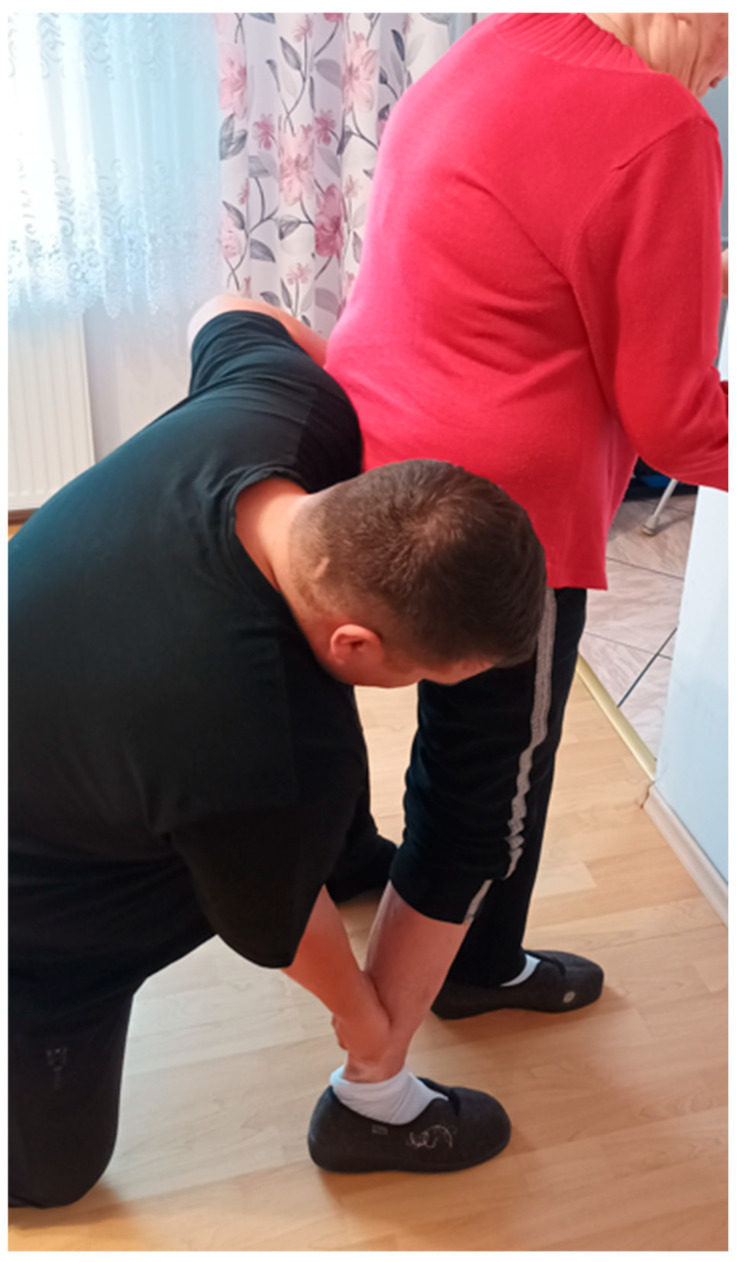
Contract-relax stretching of the triceps surae on the hemiparetic side to improve mobility in tight or shortened calf musculature, using the Contract-Relax technique from the PNF approach.

Over a two-month period, the patient received twenty physiotherapy sessions, each lasting 60 min, using PNF principles. Sessions were scheduled two to three times per week. Interventions were individually tailored and based on clinical assessment and clinical presentation. The treatment was carried out by a physiotherapist trained in the PNF approach, and supervised by a certified PNF practitioner who is recognised as an International PNF instructor. The applied PNF techniques are summarised in Table 1 and shown in Figure 1, Figure 2, Figure 3 and Figure 4.

To assess the effectiveness of the therapy, functional and standardised tests were conducted in the following order: Fugl-Meyer Assessment of Lower Extremity (FMA-LE), Trunk Impairment Scale (TIS), Timed Up and Go Test (TUG), Rivermead Motor Assessment Scale (RMA), Barthel Scale (BS), and Short Form Health Survey (SF-36). Percentage change scores were calculated relative to baseline measurements (T0).

Quality of life was assessed using the Polish version of the Short Form Health Survey (SF-36) by Tylka and Piotrowicz [19]. In this version, lower scores reflect a better perceived quality of life, in contrast to the original version by Ware and Sherbourne, where higher scores indicate a better quality of life [20]. Therefore, in this single-case study, the total SF-36 score was used as a global measure to avoid overinterpreting small intra-individual changes.

Assessments were performed at baseline (T0), after 10 session (T10), after 20 sessions (T20), and again at 3-week (T3B) and 6-week (T6B) follow-up to evaluate longer-term effects evaluate the long-term effects process. All percentage values reported in the Results section refer to changes relative to baseline (T0).

## 3. Results

A summary of patient progress is presented in Table 2.

Marked improvement was observed in lower limb motor function measured using FMA-LE. The score increased from 50 (baseline) to 54 (8%) after 10 sessions and to 59 (18%) after 20 sessions (T0–T10, T0–T20). Importantly, gains were partially maintained at follow-up: 65 points (30%) at 3-week follow-up and 60 (20%) at 6-week follow-up (T0–T3B, T0–T6B).

In contrast, trunk control measured by TIS initially declined from 14 to 12 (14.3%) after 10 sessions (T0–T10), but returned to baseline (14) at 20 sessions, and at 3-week follow-up (T0–T20, T0–T3B). A slight decline to 13 (7.1%) was noted at 6 weeks (T0–T6B).

For functional mobility, TUG performance initially worsened from 69 s to 77 s (11.6%) at 10 session, improved to 65 s (5.8%) after 20 sessions, and further improved to 63 s (8.7%) at 6 weeks.

On the RMA only minimal changes were recorded with scores returning to baseline at 6 weeks (T0–T6B).

Independence of daily living (Barthel Scale) remained stable at 85 throughout.

Finally, quality of life (SF-36) improved from 110 (baseline) to 102.5 (6.8%) after 10 sessions and to 83 (24.5%) after 20 sessions (T0–T10, T0–T20). Subsequently, after the cessation of physiotherapy, a slight deterioration occurred during follow-up, with scores of 85 (22.7%) at 3 weeks and 95 (13.6%) at 6 weeks.

## 4. Discussion

This case report highlights the feasibility and potential benefit of a PNF-based home rehabilitation program for a patient with chronic hemiplegia living in a rural area. This study presents a structured assessment framework and outlines the clinical reasoning underpinning the intervention.

A major strength of the intervention was the individualised goal-setting, tailored to the patient’s functional needs. Notably, this was the first targeted physiotherapy programme she had received since her stroke, enabling evaluation of PNF techniques without the confounding effects of previous specialised rehabilitation.

Moreover, the adaptability of the PNF approach allows its implementation in both clinical and home environments. Delivering the treatment at home improved the patient’s psychological comfort and ensured accessibility in a rural setting where transport and service availability are limited. Home-based interventions are particularly important for patients with restricted mobility who cannot attend outpatient facilities.

It is also worth noting that in neurological rehabilitation, family support and patient motivation and sustained engagement play a crucial role. Conversely, factors such as fatigue, comorbidities and loss of motivation due to prolonged recovery can hinder progress [1,9].

Importantly, the most substantial recovery after stroke typically occurs within the first three months following the event. Emerging evidence confirms that meaningful gains are still achievable in the chronic phase, particularly with targeted, task-specific interventions [21,22].

From a healthcare system perspective, home physiotherapy tends to be more costly due to travel time, reduced caseload capacity, and logistical demands [23]. However, for patients with severe mobility limitations, home physiotherapy may be cost-comparable, or even cost-saving, relative to medical transport services, which require specialised staff and equipment [24].

Additionally, access to rehabilitation in urban settings, compared to rural ones, significantly impacts treatment outcomes, improvement in neurological deficits, and Activities of Daily Living (ADLs) of stroke survivors. Patients in urban areas often benefit from better access to specialised rehabilitation centers, highly qualified personnel, modern technologies, which is associated with improved outcomes and a significant reduction in neurological deficits [25].

Evidence supporting the use of PNF in post-stroke rehabilitation continues to grow. The benefits of PNF-based treatment program, also in post-stroke patients, was presented in the work of Smedes and da Silva [26]. Although the authors of the referenced study focused primarily on improving upper limb function, they reported meaningful gains after 6 weeks of intervention (18 sessions) across multiple domains, including active and passive range of motion, grip strength, Modified Ashworth Scale, Frenchay Arm Test, Nine-Hole Peg Test, and Activities of Daily Living (ADLs). These improvements enabled the patient to perform tasks such as holding a cup of coffee and shaving. Notably, the researchers observed a slight improvement in most parameters even 4 weeks after discontinuing therapy [26].

In our study, improvement in lower limb function was demonstrated using the Fugl-Meyer Assessment of Lower Extremity Scale (FMA-LE), a widely applied and validated measure in stroke rehabilitation [27]. This improvement persisted at the 6-week follow-up, further supporting the beneficial effects of PNF techniques on hemiplegic lower limb control.

Trunk control is essential for maintaining balance against gravity, yet stroke survivors often experience impaired balance as a consequence of reduced trunk muscle strength. Thus, trunk control and balance training are key components of functional recovery in chronic stroke [28]. Reliable outcome measures are required to evaluate these processes and their impact on daily functioning and participation [29]. The Trunk Impairment Scale (TIS), known for its high reliability and validity, assesses static and dynamic sitting balance as well as trunk coordination [30]. In our study, no improvement in TIS scores was observed. This contrasts with the findings of Hwangbo et al., who reported improvements from 14.0 ± 3.4 to 18.5 ± 3.3 points in patients receiving PNF neck pattern interventions [31].

A meta-analysis by Nguyen et al. further supports the beneficial effects of PNF-based therapy on balance and gait speed [32]. These results are consistent with our observations, where improvements in gait performance were achieved and sustained for up to 6 weeks following completion of treatment. After 20 therapy sessions, the patient’s Timed Up and Go (TUG) performance improved from 69 to 65 s, with a further 2-s reduction at the 6-week follow-up. Similarly, a review by other authors confirmed the positive impact of PNF techniques on gait parameters in stroke [33]. In the study by Boob and Kovela, pelvis-focused PNF techniques improved step length, cadence, and gait speed in the 10-m walk test, as well as balance, as assessed by the Berg Balance Scale [34].

With respect to gait and balance control, patients with chronic hemiparesis appear to benefit from PNF-based rehabilitation. Improvements have been observed in gait parameters, muscle activation, and balance performance [33]. Combined PNF and treadmill training enhances both balance and walking ability, and PNF-based gait training integrated with routine physiotherapy has been shown to be more effective for improving static and dynamic balance than routine therapy alone [35]. Regarding gait parameters, PNF appears comparably effective to standard physiotherapy [36]. Preliminary studies suggest that combining PNF with advanced technologies—such as robotic gait training, electrical muscle stimulation, or kinesiotaping—may produce superior outcomes compared with single-modality approaches [37]. Other studies similarly report positive effects of PNF techniques on walking performance in stroke patients [38,39]. Collectively, PNF demonstrates a growing evidence base for improving balance, gait, and trunk control [33].

In evaluating functional recovery after stroke, systematic reviews suggest that no single neurophysiological approach—including NDT Bobath, PNF, or conventional rehabilitation—is unequivocally superior for improving upper limb function or ADL performance [40]. Some reviews indicate greater improvements following PNF-based protocols compared with conventional exercises, whereas comparisons between NDT Bobath and PNF generally show no clear advantage [41,42]. In our study, functional assessment using the Rivermead Motor Assessment (RMA) Scale revealed improvements in global, lower limb, and trunk function, but not in upper limb function. These changes were maintained at the 6-week follow-up. Comparable findings were reported by Klimkiewicz et al., who examined 30 post-stroke patients assigned to either conventional kinesitherapy or kinesitherapy combined with PNF [43]. Both groups demonstrated significant improvements in functional status and muscle tone on the Ashworth and RMA scales; however, outcomes were superior in the group receiving additional PNF.

Motor and sensory impairments following stroke significantly influence mobility, limit ADL performance, and reduce participation in social and vocational activities, thereby contributing to a reduced quality of life (QoL) [2,44]. Cognitive impairments—another common consequence of stroke—may further undermine functional independence [45].

In our study, the patient’s daily functioning remained stable, with no improvement or deterioration, retaining a score of 85/100 on the Barthel Index. However, health-related QoL, as assessed using the SF-36 questionnaire, improved substantially from 110 points at baseline to 102.5 points after 10 PNF sessions and 83 points after 20 sessions. Following discontinuation of therapy, QoL declined to 85 points at 3 weeks and further to 95 points at 6 weeks, indicating potential sensitivity to the withdrawal of treatment.

This study has several limitations. First, the intervention would have been more comprehensive had upper limb therapy and assessment also been included. Additionally, prescribing a structured home exercise programme would reflect current rehabilitation standards and may have influenced long-term outcomes. The single-case design represents a key limitation, as it restricts generalisability. Nonetheless, the findings highlight the need for further research in larger cohorts with longer follow-up periods and appropriate statistical analysis.

In summary, this case study reinforces the importance of maintaining access to physiotherapy for individuals living in rural environments—not only for stroke survivors but also for other people with disabilities. Individually tailored PNF interventions may yield meaningful benefits even several years after stroke, including when delivered in the home setting. Future research should focus on determining the optimal intensity and frequency of home-based physiotherapy to prevent deterioration associated with interruptions in treatment.

## 5. Conclusions

This single-case report describes improvements in lower limb function and gait performance in a patient with chronic stroke, as measured by the FMA-LE and TUG tests. No changes were observed in overall functional status (RMA) or independence in activities of daily living (Barthel Index). A meaningful improvement in quality of life was documented using the SF-36 questionnaire. As these findings derive from a single case, they cannot be generalised to the broader stroke population and should be interpreted with caution. Larger studies are required to evaluate the potential role of PNF therapy in chronic stroke rehabilitation, particularly in the context of home-based care in rural areas.

## Figures and Tables

**Table 1:** Physiotherapy intervention based on PNF principles.

Patient’s Position	Activity and/or PNF Pattern	Used PNF-Technique	Short-Term Goal	Purpose of the Therapeutic Application
Supine lying [Figure 1]	Flexion of non-affected lower limb joints	Combination of Isotonics	Activate the extensors of the hemiparetic lower limb	To prepare and improve the stance phase of gait on the hemiparetic side
Supine lying—bridge position	Pelvic lift/bridging	Combination of Isotonics	Strengthen the trunk and pelvic region	The enhance trunk control and improve postural function in sitting
Sitting [Figure 2]	Moving the hemiparetic lower limb forwards and backwards	Rhythmic Initiation	Teach correct movement initiation of the hemiparetic lower limb	To improve proprioception and coordination of the hemiparetic lower limb
Sitting	Trunk movements in the frontal plane	Dynamic Reversals	Improve trunk mobility and coordination	To strengthen the weaker side of the trunk and support function in sitting and gait
Sitting [Figure 3]	Trunk rotation to both sides	Combination of Isotonics	Increase trunk strength and rotational mobility.	To reduce trunk stiffness and enhance rotational control and functional strength
Sitting	Anterior and posterior pelvic tilt	Rhythmic Initiation	Improve pelvic mobility and coordination	To teach correct pelvic control required for sitting balance and efficient gait
Sitting	Trunk isometric contractions	Stabilising Reversals	Improve trunk coordination, stability, and strength	To increase trunk stability and improve functional performance in sitting and standing
Standing	Anterior elevation of the pelvis on the hemiparetic side	Rhythmic Initiation	Teach coordinated pelvic movement on the hemiparetic side	To optimise pelvic function during the swing phase of gait
Standing	Practising swing phase on the hemiparetic side	Rhythmic Initiation	Teach correct movement patterns for swing phase	To facilitate the efficient and safe swing-phase mechanics in the hemiparetic lower limb
Standing [Figure 4]	Contraction and relaxation of the triceps surae	Contract-Relax	Improve mobility of tight or shortened calf muscles	To increase ankle mobility and support functional movement at ankle and knee

**Table 2:** Detailed results of functional tests conducted before, during, and 3 and 6 weeks after the completion of 20 therapeutic sessions of physiotherapy using techniques based on the PNF concept.

	T0	T10	T20	T3B	T6B	T0–T10	T0–T20	T0–T3B	T0–T6B	T20–T3B	T20–T6B
**FMA-LE**	50	54	59	65	60	−4	−9	−15	−10	−6	−1
**TIS**	14	12	14	14	13	2	0	0	1	0	1
**TUG [sec]**	69	77	65	65	63	−8	4	4	6	0	2
**RMA**	9	8	10	10	9	1	−1	−1	0	0	1
**BS**	85	85	85	85	85	0	0	0	0	0	0
**SF-36**	110	102.5	83	85	95	7.5	27	25	15	−2	−12

**FMA-LE** = Fugl-Meyer Assessment of Lower Extremity; **TIS** = Trunk Impairment Scale; **TUG** = Timed Up and Go Test; **RMA** = Rivermead Motor Assessment Scale; **BS** = Barthel Scale; **SF-36** = Short Form Health Survey. **T0** = examination before the first physiotherapy session; **T10** = examination after 10 physiotherapy sessions; **T20** = examination after 20 physiotherapy sessions; **T3B** = examination after a 3-week break; **T6B** = examination after a 6-week break.

## Data Availability

Data supporting the findings of this study are available from the corresponding author upon reasonable request.

## Supplementary Materials

The following supporting information can be downloaded at: https://www.mdpi.com/article/10.3390/jcm14248913/s1, Table S1: TIDieR Checklist [17]; Table S2: CARE Checklist [18].
