# Supplementary material for: Home Physiotherapy Using the Proprioceptive Neuromuscular Facilitation Concept in a Patient with Chronic Hemiplegia in a Rural Area: A Case Report
Source: J Clin Med. 2025 Dec 17;14(24):8913. doi: 10.3390/jcm14248913 (PMC12734076; doi:10.3390/jcm14248913)
Supplement: Supplementary file 1 [file jcm-14-08913-s001.zip › jcm-3957430_Table_S1_TIDieR_Checklist.pdf]

TIDieR
Template for Intervention Description and Replication

Table S1. The TIDieR (Template for Intervention Description and Replication) Checklist*:

Information to include when describing an intervention and the location of the information

Item number	Item	Where located ** Primary paper (page or appendix Number/lines)	Other † (details)
	**BRIEF NAME**		
**1.**	Provide the name or a phrase that describes the intervention.<br>Home-based PNF physiotherapy in chronic stroke	1/1-4	
	**WHY**		
**2.**	Describe any rationale, theory, or goal of the elements essential to the intervention.<br>PNF is utilised in the rehabilitation of stroke patients because it enhances motor function by enhancing neuroplasticity and improving proprioceptive integration, resulting in improved movement control, muscle strength, and coordination[5]. Importantly, the most substantial recovery after stroke typically occurs within the first three months following the event. Emerging evidence confirms that meaningful gains are still achievable in the chronic phase, particularly with targeted, task-specific interventions[19,20].<br>Moreover, the adaptability of the PNF approach allows its implementation in both clinical and home environments. Delivering the treatment at home ensure accessibility in a rural setting where transport and service availability are limited. Home-based interventions are particularly important for patients with restricted mobility who cannot attend outpatient facilities. PNF-based physiotherapy delivered at home environment may provide meaningful benefits for patients living in rural areas, even years after stroke.<br>Therefore, the study aimed to assess the impact of selected PNF techniques on a chronic stroke patient during home physiotherapy in rural areas.	2/43-45; 7/178-181; 6-7/169-173; 1/29-30; 2/70-71	

	**WHAT**		
**3.**	Materials: Describe any physical or informational materials used in the intervention, including those provided to participants or used in intervention delivery or in training of intervention providers. Provide information on where the materials can be accessed (e.g. online appendix, URL). No physical materials or devices were used. The intervention relied exclusively on therapist-applied manual PNF techniques. Procedural details are provided in Table 1 and Figures 1–4.	2/57-59; 3-5/102-115	
**4.**	Procedures: Describe each of the procedures, activities, and/or processes used in the intervention, including any enabling or support activities. A detailed description of the applied PNF techniques is provided in Table 1 and Figures 1–4.	3-5/102-115	
	**WHO PROVIDED**		
**5.**	For each category of intervention provider (e.g. psychologist, nursing assistant), describe their expertise, background and any specific training given. The treatment was carried out by a physiotherapist trained in the PNF approach, and supervised by a certified PNF practitioner who is recognised as an International PNF instructor.	3/98-100	
	**HOW**		
**6.**	Describe the modes of delivery (e.g. face-to-face or by some other mechanism, such as internet or telephone) of the intervention and whether it was provided individually or in a group. Therapy using PNF techniques was performed individually by a physiotherapist.	3/97-98	
	**WHERE**		
**7.**	Describe the type(s) of location(s) where the intervention occurred, including any necessary infrastructure or relevant features. The rehabilitation program was delivered in the patient's residence within a rural community in the Pomeranian Voivodeship in Poland.	3/92-94	

	**WHEN and HOW MUCH**		
**8.**	Describe the number of times the intervention was delivered and over what period of time including the number of sessions, their schedule, and their duration, intensity or dose.	______	______
	Over a two-month period, the patient received twenty individual physiotherapy sessions, each lasting 60 minutes, using PNF principles. Sessions were scheduled two to three times per week.	3/95-97	
	**TAILORING**		
**9.**	If the intervention was planned to be personalised, titrated or adapted, then describe what, why, when, and how.	______	______
	Interventions were individually tailored and based on clinical assessment and clinical presentation.	3/97-98	
	**MODIFICATIONS**		
**10.‡**	If the intervention was modified during the course of the study, describe the changes (what, why, when, and how).	______	______
	N/A		
	**HOW WELL**		
**11.**	Planned: If intervention adherence or fidelity was assessed, describe how and by whom, and if any strategies were used to maintain or improve fidelity, describe them.	______	______
	N/A		
**12.‡**	Actual: If intervention adherence or fidelity was assessed, describe the extent to which the intervention was delivered as planned.	______	______
	N/A		

** **Authors** - use N/A if an item is not applicable for the intervention being described. **Reviewers** – use '?' if information about the element is not reported/not sufficiently reported.

† If the information is not provided in the primary paper, give details of where this information is available. This may include locations such as a published protocol or other published papers (provide citation details) or a website (provide the URL).
‡ If completing the TIDieR checklist for a protocol, these items are not relevant to the protocol and cannot be described until the study is complete.

* We strongly recommend using this checklist in conjunction with the TIDieR guide (see *BMJ* 2014;348:g1687) which contains an explanation and elaboration for each item.

* The focus of TIDieR is on reporting details of the intervention elements (and where relevant, comparison elements) of a study. Other elements and methodological features of studies are covered by other reporting statements and checklists and have not been duplicated as part of the TIDieR checklist. When a **randomised trial** is being reported, the TIDieR checklist should be used in conjunction with the CONSORT statement (see www.consort-statement.org) as an extension of **Item 5 of the CONSORT 2010 Statement.** When a **clinical trial protocol** is being reported, the TIDieR checklist should be used in conjunction with the SPIRIT statement as an extension of **Item 11 of the SPIRIT 2013 Statement** (see www.spirit-statement.org). For alternate study designs, TIDieR can be used in conjunction with the appropriate checklist for that study design (see www.equator-network.org).
