# Supplementary material for: Home Physiotherapy Using the Proprioceptive Neuromuscular Facilitation Concept in a Patient with Chronic Hemiplegia in a Rural Area: A Case Report
Source: J Clin Med. 2025 Dec 17;14(24):8913. doi: 10.3390/jcm14248913 (PMC12734076; doi:10.3390/jcm14248913)
Supplement: Supplementary file 1 [file jcm-14-08913-s001.zip › jcm-3957430_Table_S2_CARE_Checklist.pdf]

CARE case report guidelines

Table S2. CARE Checklist of information to include when writing a case r[illegible]

(cc) BY-NC-ND

Topic	Item No	Checklist item description	Reported on Page Number/Line Number	Reported on Section/Paragraph
Title	1	The diagnosis or intervention of primary focus followed by the words “case report”	1/1-4	Title
Key Words	2	2 to 5 key words that identify diagnoses or interventions in this case report, including "case report"	1/31-33	Keywords
Abstract (Structured summary)	3a	Background: state what is known and unknown; why the case report is unique and what it adds to existing literature.	1/15-20	Abstract
	3b	Case Description: describe the patient’s demographic details, main symptoms, history, important clinical findings, the main diagnosis, interventions, outcomes and follow-ups.	1/20-29	Abstract
	3c	Conclusions: summarize the main take-away lesson, clinical impact and potential implications.	1/29-30	Abstract
Introduction	4	One or two paragraphs summarizing why this case is unique **(may include references)**	1-2/39-53	Introduction
Patient Information	5a	De-identified patient specific information	3/89-91	Material and Methods
	5b	Primary concerns and symptoms of the patient	2/76-78	Material and Methods
	5c	Medical, family, and psycho-social history including relevant genetic information	2/78-79	Material and Methods
	5d	Relevant past interventions with outcomes	7/166-168	Discussion
Clinical Findings	6	Describe significant physical examination (PE) and important clinical findings	2/84-88	Material and Methods
Timeline	7	Historical and current information from this episode of care organized as a timeline	3/95-97	Material and Methods
Diagnostic Assessment	8a	Diagnostic testing (such as PE, laboratory testing, imaging, surveys).	5-6/116-130	Material and Methods
	8b	Diagnostic challenges (such as access to testing, financial, or cultural)	N/A	
	8c	Diagnosis (including other diagnoses considered)	2/89-91	Material and Methods
	8d	Prognosis (such as staging in oncology) where applicable	N/A	
Therapeutic Intervention	9a	Types of therapeutic intervention (such as pharmacologic, surgical, preventive, self-care)	3-6/102-115	Material and Methods
	9b	Administration of therapeutic intervention (such as dosage, strength, duration)	3/95-97	Material and Methods
	9c	Changes in therapeutic intervention (with rationale)	N/A	

Follow-up and Outcomes	10a	Clinician and patient-assessed outcomes (if available)	6/142-159	Results
	10b	Important follow-up diagnostic and other test results	6/134-141	Results
	10c	Intervention adherence and tolerability (How was this assessed?)	N/A	
	10d	Adverse and unanticipated events	N/A	
Discussion	11a	A scientific discussion of the strengths AND limitations associated with this case report	7/165-177; 9/263-269	Discussion
	11b	Discussion of the relevant medical literature **with references**	8/203-256	Discussion
	11c	The scientific rationale for any conclusions (including assessment of possible causes)	7/174-177; 7-8/193-207	Discussion
	11d	The primary “take-away” lessons of this case report (without references) in a one paragraph conclusion	9/278-285	Conclusions
Patient Perspective	12	The patient should share their perspective in one to two paragraphs on the treatment(s) they received	N/A	
Informed Consent	13	Did the patient give informed consent? Please provide if requested	**Yes** ☒	**No** ☐

Please leave this space alone as it will be supplemented by the editorial office when needed.
